# Orymold: ontology based gene expression data integration and analysis tool applied to rice

**DOI:** 10.1186/1471-2105-10-158

**Published:** 2009-05-23

**Authors:** Jaume Mercadé, Antonio Espinosa, José-Enrique Adsuara, Rosa Adrados, Jordi Segura, Tamara Maes

**Affiliations:** 1 Oryzon Genomics, Parc Científic de Barcelona, 08028 Barcelona, Spain

## Abstract

**Background:**

Integration and exploration of data obtained from genome wide monitoring technologies has become a major challenge for many bioinformaticists and biologists due to its heterogeneity and high dimensionality. A widely accepted approach to solve these issues has been the creation and use of controlled vocabularies (ontologies). Ontologies allow for the formalization of domain knowledge, which in turn enables generalization in the creation of querying interfaces as well as in the integration of heterogeneous data, providing both human and machine readable interfaces.

**Results:**

We designed and implemented a software tool that allows investigators to create their own semantic model of an organism and to use it to dynamically integrate expression data obtained from DNA microarrays and other probe based technologies. The software provides tools to use the semantic model to postulate and validate of hypotheses on the spatial and temporal expression and function of genes. In order to illustrate the software's use and features, we used it to build a semantic model of rice (*Oryza sativa*) and integrated experimental data into it.

**Conclusion:**

In this paper we describe the development and features of a flexible software application for dynamic gene expression data annotation, integration, and exploration called Orymold. Orymold is freely available for non-commercial users from

## Background

The number of completely sequenced genomes is rapidly growing as sequencing costs decrease. Sequence information, the availability of genome and proteome wide expression monitoring techniques such as DNA microarrays (MA), and established molecular biology techniques like *in situ *hybridization (ISH) are crucial in the understanding of the genetic complexity of organisms. Laser Capture Microdissection (LCM) [1] and Fluorescent Activated Cell Sorting (FACS) are increasing gene expression monitoring resolution to the cellular level [2,3]. Given the ever increasing quantity and multidimensionality of the data produced by the '-omics' era, it has become a main objective for bioinformaticists to produce software that allows data analysis and storage. To date, the best approach for solving generalization in data query interfaces and integration of data has been the development of controlled vocabularies (ontologies) [4,5]. Ontology construction has been a hot topic for several years in the bioinformatics field since it provides a rich framework for dynamic annotation, sharing of data, and formalization of domain knowledge. Ontology properties promote the creation of comprehensive views and comparisons of data both on a human and machine comprehensive basis.

Several groups have pursued the development of biological organism centred and biologically comprehensive tools capable of dynamically integrating heterogeneous experimental data under a common model. Initiatives such as the Plant Ontology Consortium [6] have already begun working on the design of an ontology to describe the distinct structures, growth, and developmental stages of plants. This will provide a framework for meaningful integration of experimental and phenotype data and allow cross-species queries across databases.

Some web-based applications already provide curated experimental data integrated in or annotated using an ontology describing a part or a whole organism [7-9]. Such applications provide extensive experimental data repositories and tools for analysis and gathering of existing data. However, generally these lack that degree of flexibility necessary for integration of new data in a customized manner, necessary when experimenting *in silico*. To our knowledge there is no biological software available yet that provides both tools for gene expression data integration and visualization and means to dynamically modify the data integration structure so new proposals on data organization can be formalized.

In this paper we describe the functional objectives and features of Orymold, a desktop software for the contextual management of gene expression data. We have designed the application to be flexible enough to be able to include new terms in the ontology and thus rapidly mold it to accept new data. We briefly describe the technical aspects of the implementation and demonstrate the features of Orymold by building a semantic model of the model organism *Oryza sativa*. Next we illustrate how the model can be used to include macro and microscopic pictures of organs, tissues, and cell types and to integrate gene expression data.

## Implementation

### Orymold client and experimental database

**Figure 1:**
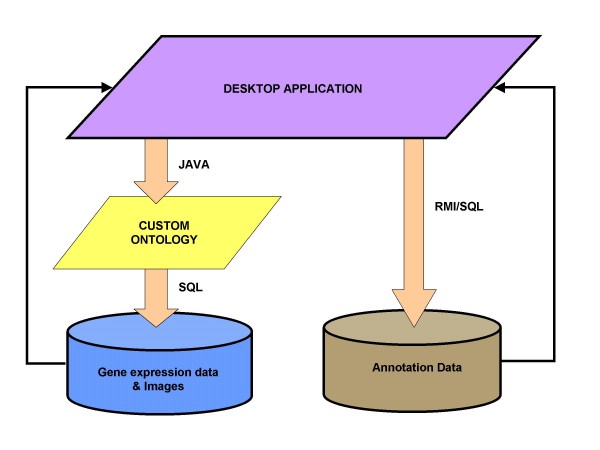
**Conceptual diagram of the building blocks of Orymold**. From the Java Swing interface of the application, users can access the ontology and the gene expression data, annotations, and images linked to it and stored in the underlying DBMS. The ontology serves simultaneously as an interface between data and user and as integration scaffold, allowing for the construction of semantic queries.

We designed Orymold following what is known as two tier architecture: a standalone client is in charge of data processing and presentation while a relational database management system (RDBMS) manages experimental data storage and retrieval (Fig. 1). This type of architecture allows for concurrent and remote access to many users in a scalable way, and it provides for the distribution of data. The open source MySQL 5.0 [10] RDBMS was chosen to store both genomic and experimental data. MySQL provides a fair trade-off between enterprise level performance and reasonable administration requirements. Client database connectivity is managed through MySQL proprietary JDBC driver.

We chose the Java SE 6 programming language [11] to implement the client, thus ensuring portability through most operating systems while taking into account future application extension. Another advantage is that we do not have to rely on frameworks; only established technology is used. The application has been successfully tested on Microsoft Windows XP and on Linux. Java was chosen because of its powerful libraries on GUI construction such as Swing and on image processing such as Java Advanced Imaging. In order to enable database access, Java Database Connectivity (JDBC) application programming interface (API) was employed.

### OryDB architecture

**Figure 2:**
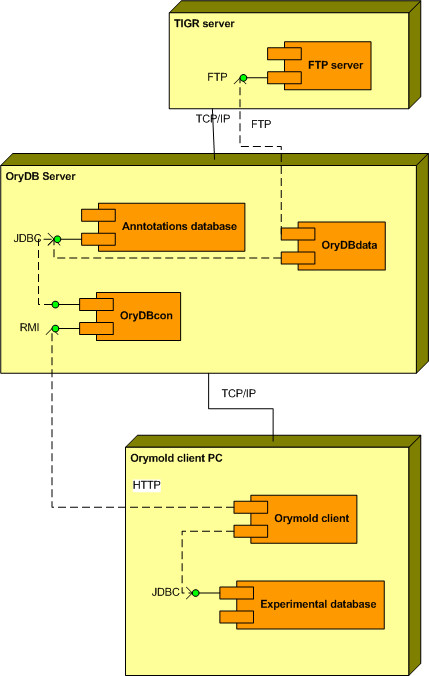
**Unified Modelling Language (UML 2.0) deployment diagram of Orymold system**. Orymold is a distributed system composed of a client and an annotations database server. The annotations server, OryDB, stores annotations from TIGR using its publicly available FTP server through the OryDBdata component. The OryDBcon component from OryDB provides a web service for retrieving annotations and mapping sequences as requested by the Orymold remote client. The Orymold client component communicates with the OryDB system through its RMI interface, and manages data stored in the experimental database. It has to be noted that the experimental database could be distributed and serve as a common experimental data warehouse for several clients.

OryDB is organized in two layers: a persistence layer and a management and connection layer. The persistence layer is implemented using a RDBMS that allows for relational organization of the information and easy customisation of indexes for fast query, retrieval, and parsing of the results. We developed a Java code layer to manage and process data, populate and update the database, perform remote BLAST [12], and provide fast connection and extension with the Orymold client through web services implemented using the Java Remote Method Invocation (RMI) API (see Fig. 2). Thus, OryDB can be considered an autonomous system providing annotations through its web services interface.

### Atlas construction

#### Tissue processing and microscopy

For microscopic pictures, plant fragments were fixed overnight (O/N) in FAA solution (50% ethanol, 5% acetic acid, 4% formaldehyde) and dehydrated in an ethanol series. The ethanol was replaced gradually by xylene. Paraplast chips were added and allowed to slowly dissolve O/N in the solvent. The solvent was then replaced by molten paraplast and the plant fragments were incubated several days at 65°C prior to being transferred to small cassettes in which they were allowed to solidify. The cassettes with the tissue fragments were cooled to 4°C, mounted onto a microtome, and cut into ribbons of 10 μm thick sections, which were mounted on 3-amino-propyltriethoxy silane coated slides. Slides containing samples of interest were selected using a fluorescence microscope, incubated in a xylene-ethanol dilution series, and rehydrated in an ethanol-water dilution series. The sections on the slides were stained using 0.5% calcofluor white or propidium iodide in 0.1 M arginine buffer pH 12.4 or propidium iodide. Images of the sections were taken using a Leica SP2 confocal microscope using a 351 nm light source and a 364 nm filter (calcofluor white) or a 543 nm filter (propidium iodide). Depending on the tissue, either one or the overlay of both images was used in the atlas.

## Results

### Overview of Orymold

Orymold is a software application composed of an annotations server (OryDB) and a thick client with an experimental data database (Fig. 2). The annotation server is in charge of storing public annotation information while the client's experimental database is used to store gene expression data from both single and batch experiments. The client provides a friendly graphical user interface (GUI) that allows for the retrieval of stored annotation and gene expression data through a user modifiable ontology. The client uses the ontology to present data to the researcher, allowing him or her to interactively create views by semantically querying the data (Fig. 1). With the ontology, the client provides for the creation of an ontology coherent atlas of pictures, which enhances the visualization and the biological contextualization of both the ontology and the data.

Given the modular design of the system, different possible configurations are available to make it more adaptable to user needs. The system can be installed as a personal tool on a single computer, or it can be distributed to serve as a common tool for several researchers across different computers and/or servers [see Additional file 1].

### OryDB, the annotation server

OryDB automatically downloads, stores, and processes public genomic sequence information and annotations from TIGR rice genome annotation database [13,14]. Using TIGR's gene loci as a reference feature for univocal identification between databases, we integrated annotations from the Plant Proteome Annotation Program [15] and RAP-DB [16,17] as well as its links to Gene Ontology [4] and InterPro [18]. Other sources of sequence annotation based on TIGR loci can also be easily included using General Feature Formats GFF [19] and GFF3 [20] file formats when updating the database. We designed a generic annotations table to accept most usual sources of annotation information in order to make the data model easily expandable. Such design approach avoids having to create new customized tables or redesigning the currently existing ones when more sources become available, thus reducing the impact on client applications relying on it. From this point of view, OryDB should not be considered only as a pure database system that serves answers to queries generated by multiple clients, but as a data subsystem designed to embrace the expected future evolution of biological data, extending the organism original data models over time. In addition to this, using well established sources of annotation information has several advantages, such as being able to integrate any tool suitable for the data stored, like TIGR's genomic visualization tool Rice genome browser[21], which is fully integrated in Orymold client.

### Orymold client

The Orymold client design principles are to present the user a tool to query a rich set of gene expression experiments using a semantic and visual description of the complexities of an organism. The description of the organism provides an scaffold where to link both experimental and graphical data, thus allowing for the construction of a set of metadata enhanced methods for querying such data. The client provides methods for inserting, browsing, deleting and modifying the ontology, and for the creation and navigation of the atlas of organism pictures.

We designed the user interface to ensure easy access and retrieval of stored data while maintaining a constant biological contextualization in its presentation.

The main GUI of the Orymold client is organized in three parts: a browsable tree that displays the ontology describing the organism, an interactive atlas of pictures linked to the terms in the ontology, and two data containers that allow for the creation of lists of experimental features and regions of interest. All parts of the user interface are interactive and/or navigable, and they possess contextual menus that define and allow for the operations that can be performed. From the menu bar in the main interface, investigators can set which kind of data they are going to work with (MA or single probe hybridization based methods), set expression thresholds, and access DB administrative tools.

We describe main features and options of the Orymold client in the following sections.

### The ontology, a scaffold for data integration and querying

Orymold ontology contains more than three hundred terms describing the anatomy and development of *Oryza sativa *and is essentially based on the ontology of Gramene [22-24], although the hierarchy was adapted to the visual context of the atlas (see next section). The description includes a repertoire of cells and tissue types represented in the different maturation stages of the organs of the rice plant. The ontology is constrained to a single relation between terms: each child term is considered to be "part of" its parent term. Such hierarchical organization provides for easy interrelation with the atlas of pictures (as will be discussed in the next section), where pictures will display or represent a term and contain a group of child terms. Although there is no metadata stored to define the relation between terms, the "is a part of" relation is used when retrieving and performing operations on data. Hereafter, we will use "term" when referring to a node (as in graph theory) of the ontology tree, and "region" (of the organism) when referring to the actual organ, tissue, or cell type represented by such node.

The ontology can be modified and new terms can be introduced to adjust it to the particular vision or experimental design of the user. For example, *in situ *hybridization experiments can define subpopulations of cells predestined to evolve to a specific cell type even before these cells show morphological differences with respect to their neighbouring cells. These precursor cells can be conceptually represented in the ontology. Modifications of the ontology and insertion of new data can be performed by means of a contextual menu on the ontology tree. Experimental data can be linked (and thus annotated) to any term in the ontology and accessed from both the ontology tree and the lists.

### Enhancing biological contextualization using an atlas

**Figure 3:**
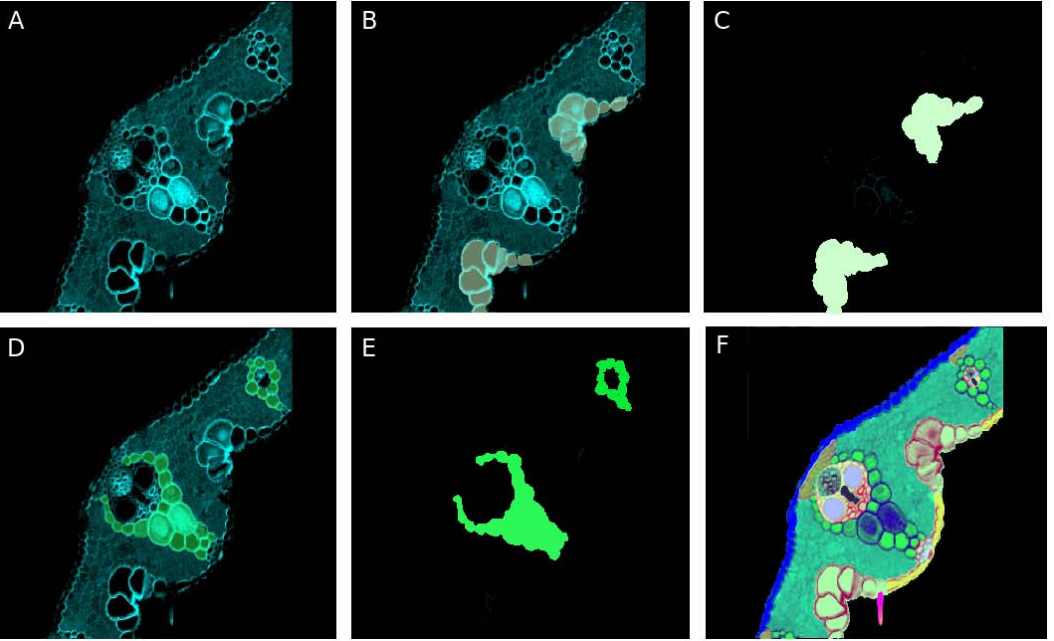
**Creation of a map for the transversal section of a rice leaf**. A. Confocal Image of a rice leaf section. B. Using image processing software, the leaf section image is divided into two layers, and the region occupied by bulliform cells is colored. C. The map of bulliform cells for the leaf section is obtained by saving the colored layer as a different archive. D, E. By repeating the process described previously in B and C, the map for the bundle sheath region is obtained. F. By overlaying all obtained maps for all regions identified in the leaf section, we can construct the full map for the picture, and when overlaid with the original picture, each region becomes distinctively painted in a different color.

We designed and implemented the atlas module to permit any kind of picture, collage of pictures, drawings or schemas, and to properly relate them to any ontology term. The main characteristic of this atlas is that it can be easily constructed from a picture by processing it with any image processing software. The only prerequisite for suitability of a picture is on a conceptual level and requires that all pictures related to a term of the ontology be fully coherent with it; the picture has to globally represent the term and all the child terms of such term individually. For instance, based on the ontology of Gramene, we described the term "flower" as containing eight child terms: carpel, stamen (and their stages), glume, lemma, palea, radicle, sterile lemma, and lodicule. Thus the chosen picture to represent "flower" has to show all the parts of the flower. The child terms chosen for a picture are best chosen at the same hierarchical level, and represent similar plant regions -i.e. organs, tissues or cell types, without mixing types. When this condition is fulfilled by choosing an appropriate picture, the curator can divide the picture in as many layers as child terms are identified in the picture and paint each identified region in a distinct colour, one per layer (see Fig. 3). Once picture layers have been properly processed, they are integrated into the application together with the original picture, where they are merged in an interactive map that permits explanatory visualization of the biological term and its child terms in the ontology. Users can turn on a 'map mode' that will allow them to highlight and name the different regions (represented in the ontology by the child terms) present in the picture as they move the mouse pointer over it (see Fig. 3). A complete map with all regions highlighted in different colours is available as well. This application feature allows for a fast biological contextualization and explanation of the relations of the terms within the ontology and provides a powerful educational tool for those not familiar with the organism. For the moment, the deepest level of information available is the cellular level, but data relative to sub-cellular localization could be readily integrated. This would be particularly useful for the incorporation of protein localization data.

### Expression data in the experimental database

We designed the experimental database to store data from both individual (single gene) or batch (whole genome) experiments. All expression data, whether produced individually or *en masse*, are entered with relation to the basic information unit represented in Orymold: the gene. As such, data types that can be introduced into Orymold include *in situ *hybridization, promoter fusion lines, immunohistochemistry (IHC) data, and, as long as the insertion sequence is known, data of tagged reporter lines.

Each probe sequence included in an experiment is mapped by means of BLAST to a corresponding gene stored in the OryDB database at insertion time. From this point on, all annotations stored in OryDB related to that gene become available automatically for the probe and are therefore accessible through Orymold. Links between genes and probes are updated every time OryDB is updated, resulting in a time dependent refinement and evolution of the experiments as genomic knowledge grows or as annotation errors are corrected. Storage of data associated with the exact probe sequence rather than with the gene name or identifier is also necessary as classical gene expression analysis moves on to interpret the presence of splice variants.

### Data insertion

Orymold provides two user privilege modes: standard users and curators-administrators. This role distinction allows collaborative initiatives to keep the model controlled and to ensure data consistency. Curators are allowed to modify the model as well as to insert experimental data, while users can only browse and perform queries on the available data. Orymold is organized in two distinct data modes: MA transcriptomic studies and single probe-based expression detection. Coherent interfaces for each data type have been implemented for easy insertion and retrieval. Transcriptomic and *in situ *hybridizations probe sequences are automatically mapped by means of BLAST to the OryDB database at insertion time. The application has been implemented to accept tabulated plain text files for batch insertions (DNA MA) and pictures in Joint Photographic Experts Group (JPEG) format for experimental evidence in visual format (single probe hybridization methods).

#### Single probe data

Single probe based data, for instance *in situ *hybridizations, generally come in the form of experimental pictures. Usually this kind of information is released as a limited selection of the nicest pictures in a scientific publication, resulting in a loss of valuable information. Orymold is able to store and retrieve single probe experimental data in the following way: when an *in situ *hybridization experiment is inserted, Orymold allows the user to upload the experimental picture in the hierarchical level that is suitable for the experiment definition. In addition to this, the user can manually insert specific levels of expression of the probe corresponding gene for the ontology levels where expression has been observed. From this data description, Orymold client automatically builds a qualitative interpretation and representation of the expression using the corresponding picture of the atlas and its maps. Therefore, both the original image and the experiment representation can be retrieved; however, and importantly, the totality of the qualitative representations in the application can be submitted to semantic searches, as will be explained in a following section.

**Figure 4:**
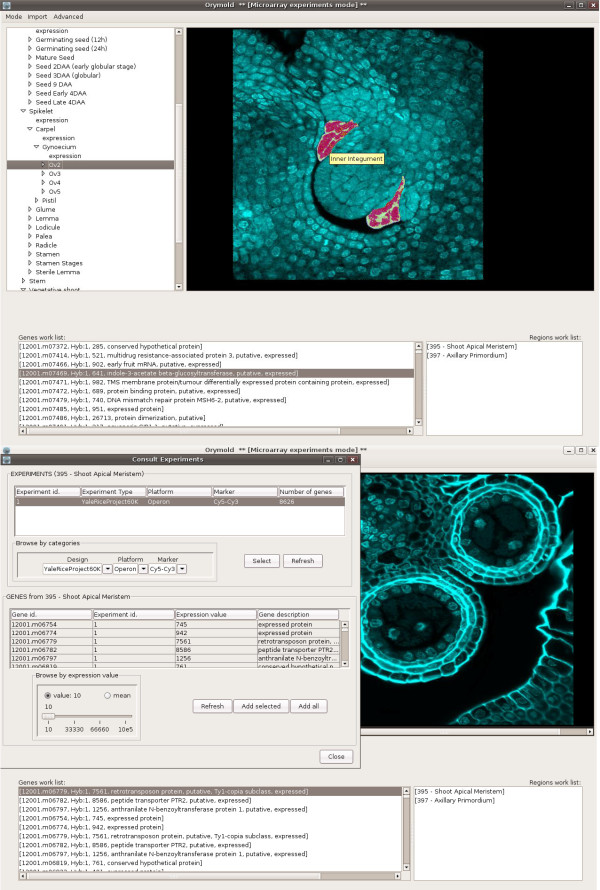
**Orymold interfaces**. Upper image shows the main interface of the application, with the customizable ontology on the upper left part, the atlas on the upper right, and the working lists at the bottom. The inner integument region of the ovule on the atlas picture appears highlighted as a result of sliding the mouse pointer over it, allowing for a fast and visual identification of each region. Bottom image shows the basic interface for retrieving MA data from a selected region. Users are able to browse experiments and its contained data. In the background, the atlas feature shows a section of an anther.

Single probe data insertion involves the mapping of a single probe sequence to a sequence present in the OryDB database, as well as the insertion of the experimental picture where the expression of the targeted mRNA is evidenced. Since the experimental picture will probably show expression of the probe target for more than a single term in the ontology, the probe will have to be introduced in the higher term in the hierarchy represented in the picture. By selecting such term in the ontology and selecting "insert data" in the contextual menu, a pop-up interface will appear, allowing the investigator to upload and define the data to insert (see Fig. 4). The insertion interface allows for the relation of a probe sequence in FASTA format with an experimental picture. All child terms of the term where the investigator has chosen to insert data are listed to provide the quantification of the expression detected by the probe for each of them. The qualification will be based in the visual interpretation of the experimental picture by the researcher-curator in a discrete range from zero to four. Since it is possible that a listed term will not appear in the picture or be unclear as to whether there is expression or not for a given region, a "N/A" qualification is allowed for the term to indicate that there is no conclusive evidence for expression or for non-expression. Along with the probe sequence, the expression values for all terms, and the experimental picture, an atlas based composition will be created and stored according to the expression values. Using the parent term to retrieve the main picture and the coloured maps of the regions set as having expression (one to four in the discrete range) by the curator, a composition will be automatically created and stored at insertion time, taking full advantage of the atlas feature. Thus, the system will be storing two visual resources of the experiment: the original picture of the experiment and the fully atlas integrated representation of the expression as interpreted by the curator.

#### Microarray data

While the previously discussed data are qualitative, or at the most semi-quantitative, we also designed Orymold to deal with quantitative data derived from the MA analysis of different organs, cell types, varieties, or mutants. Orymold allows for the integration of expression data obtained on any MA technological platform (including single colour and dual colour experiments), although evidently the expression values of different MA platforms cannot always be expected to yield directly comparable information. To maximize the cross-comparability of dual colour data, we advise using a reference sample in each set of MA experiments, normalizing the data of the other samples to the reference sample prior to the introduction of the data into Orymold, and applying strict criteria on data quality prior to admitting them for storage. In the current build, we introduced thirty-four MA experiments performed by Prof. Nelson's research group for the RICEATLAS Project [25].

When dealing with batch insertions like MA data, the insertion method is divided into two steps. First, the design file of the MA has to be introduced in the database. That step implies the mapping of all the probes in the design to their target sequences in the OryDB database by means of BLAST, which can be time consuming depending on the number of probes in the design. Second, once the design exists in the experimental database, expression data can be related to it: users select the design and upload the expression data file. Introduction of expression data from a 60 K design using the same machine over the same Local Area Network (LAN) takes only a few minutes.

Once data has been inserted, consultation is straightforward. By clicking on a term in the ontology tree, the user will access all data linked to that term. Depending on the selected data mode, a different interface will pop up, enabling the user to browse the distinct experimental results.

### Data exploration

#### Microarray data

When retrieving MA data, the user is presented with a pop-up interface with two browsable tables (see Fig. 4). The upper table allows the user to browse through available hybridizations for that region. Hybridizations can be rapidly filtered using parameters such as MA platform (i.e. 60 mer), design, and sample labelling. Once a set of parameters have been selected, the list of hybridizations can be reloaded, presenting the user with the parametrized result. Then a hybridisation can be selected and loaded into the lower table. From the start, all probes with their primary matching annotations and expression values will be listed. The user can shorten the resulting list by using a slider that sets up a lower threshold for the experimental values. If the user finds interesting results, those can be sent to the work list in the main interface for further consultation. Once in the list, any gene can be selected and inquiries can be made about its expression. A list of all regions of the organism having the gene expressed will be listed in the regions list in the lower left side of the main interface. Since two or more terms can be named the same as long as at least one of the parent terms is not shared, we have implemented a tree path consultation feature. This allows users to list (in order) all the parent terms in the path from the root of the tree to a selected term, therefore the user can indistinctly identify each term in the work list. Given that expression data is mapped to that of TIGR's, Orymold is enabled to show the genomic annotations by opening a web browser and taking advantage of TIGR's Rice Genome Browser.

#### Single probe data

Retrieving single probe-based data from a chosen term in the ontology is simple: the user selects the term and retrieves the data with a mouse click in the corresponding option of the contextual menu. The related data will appear instantaneously listed in the work list and show the expression and the annotation obtained from OryDB. Once there is data in the list, the genes can be interrogated about their spatial and temporal expressions across the organism the same way as in MA experiments. Moreover, the stored visual data can be retrieved for each gene in the list. Selecting a gene will allow users to retrieve both the full size and a zoom enabled original experimental picture and to browse the atlas based compositions constructed at insertion time.

### Semantic querying of the experimental database

Semantic queries allow users to ask questions like: "Where in my model does gene (X) get expressed?" When the model is linked to quantitative data, semantic queries can be extended to: "Where does gene X get expressed over a certain threshold (T)?" Or, "Which genes get expressed in the range of (value1)-(value2) in two selected (tissues, organs or cell types)?" In the opposite direction, the researcher can perform questions such as, "Which region(s) of the organism show expression for gene 1 and for gene 2 over a certain expression threshold (T)?" In-depth analysis of experimental data is beyond the scope of this work; instead, we focus on validating the data itself and the model, and we try to point out possible trends in the data that may be identified by spatial and/or temporal distribution across the organism.

The researcher has access to the semantic querying feature in the menu once data has been retrieved from the ontology tree and it is listed in the work list in the lower part of the main interface. Selecting any two genes or regions in the lists, the researcher will be able to perform semantic queries by using Boolean logic operators. The logic operators we have made available are AND, OR, and NOT. The global expression threshold applies when performing such queries, allowing for the full advantage of the operator NOT since those probes with expression values under the threshold will not be considered. Basic queries are available for all data modes, although the results are slightly different depending on the mode. In single probe mode, when a gene is found to be expressed in a region represented by a term, it will also be reported to be expressed for all the parent terms. For instance, that implies that when retrieving the expressed regions for a stamen specific gene, "stamen", "flower" and "spikelet" will all be retrieved. It also implies that when retrieving the genes expressed in "spikelet", we will also retrieve the genes we have expression evidence for in "stamen". In MA mode, on the contrary, that would lead to the retrieval of such an amount of data that the query was restricted to the single selected term -i.e., only the expression data for "spikelet" would be retrieved.

More complex queries are available for the MA data mode from the menu bar of the main interface. Such complex queries allow for the further refinement of the parameters of the basic queries. When the complex query is selected, a new interface pops up, which allows the user to set up a group of parameters. In basic semantic queries, the parameters apply equally to both genes and regions in the operation, while in complex queries each term of the operation has its own set of parameters. For complex queries, the researcher can set up new expression thresholds that will override the global one. For instance, the researcher is allowed to ask: "Which regions of the organism fulfil the requirement that (gene 1) is expressed above (value1) AND (gene 2) is expressed below (value2)?" Or, vice versa: "Which genes are expressed above (value 1) in (region 1) AND are expressed below (value 2) in (region 2)?" These kind of queries are especially effective when trying to point out specificities of genes in close cell types or functional relations between organs or tissues. After setting a threshold, the user is allowed to set up a range of expression (value 1.1 -value 1.2) for each of the genes or regions involved in the operation.

Another useful operation for MA data is to compare regions of the organism in terms of factors of the expression of genes. From the same menu bar, the user can select to "operate by factors," and a new interface will pop up. This interface provides a forum to compare two regions in the following manner: "Which genes are (four times) more expressed in (region 1) than in (region 2)?"

In both cases, whether using complex queries or operating with factors, the result is presented in a new pop-up interface that contains the list of genes or regions meeting the query parameters. Those results can be browsed and imported back to the main interface to contribute in creating refined lists of interest to the researcher [see Additional file 2].

## Discussion

To date, bioinformatics consists of many different mainstreams.

On one hand, algorithms are developed to define what should be measured (probe design, calculation of signal intensity) or to statistically treat and compare the experimental measurements. These algorithms, independently of their implementation framework, generally work really well to generate valid data within a multidisciplinary team in a research lab.

On the other hand, the amount of information available is steadily growing, thus becoming increasingly difficult to retrieve, analyse and compare. This problem can only be really solved if the original information is organized in a different manner in the first place.

Bio-ontologies are being developed to rationally define organisms and biological systems, its alterations (disease, infection) and its interaction with the environment (heath, cold, contamination) in a structured and intuitive manner so that information can be organized and easily retrieved.

Since data retrieval is critical, text recognition algorithms have also been developed to try to semantically organize and interpret published results *a posteriori *in a fully or semi automated way, but these methods are highly error prone.

It is still an open challenge for bioinformatics to build easy-to-use tools able of rapidly and interactively integrate data with a semantic model. In general, data interaction models haven't been refined yet.

In this paper we described Orymold, a flexible software application designed for the semantic exploration of heterogeneous experimental data. Orymold consists on the OryDB server, which contains an annotations database, and the Orymold clients, which contain an experimental database, an ontology of the rice plant and a corresponding atlas of annotated pictures to visually represent the organism. Experimental data integrated into Orymold can be submitted to semantic queries.

Our approach to cope with experiment evolution over time has been to build a tool with which users (researchers) can dynamically build their own semantic semi-formal model and thus integrate new data with each new ontology element added. This is an improvement over current semantic tools, which force researchers to use existing rigid ontologies to map their data to available models. For instance, that is the case of analysis tools based on GO, where usually the data is characterized according to the three distinct ontologies (molecular function, cellular localization, and associated biological process), which are pre-fixed. The result of these analyses is a static annotation of the dataset, which has to be repeated each time GO is updated.

The modelling of ontologies and their integration with experimental data can be generally accomplished using the combination of a general purpose ontology editor and a mathematical programming language. By contrast, Orymold GUI has been designed having in mind non computer literate users. Orymold merges all functionalities under a single, common interface that provides easy and intuitive graphical tools for the construction of ontologies and posterior biological data integration.

Using Orymold, new experimental data will be automatically contextualized and ready to be analysed together with data previously integrated in the model -i.e. New gene expression experiments performed on uncharacterised cell types will be readily integrated with existing data by just adding the needed new terms in the ontology. Orymold provides an environment for the full cycle of experimental data: integration, exploration which may suggest new experiments, modification of the integration scaffold to allow the introduction of new data, and back to the beginning, when new data is inserted. Probe annotations evolution over time is also ensured by the OryDB sequence-based updating feature.

Orymold represents the first step in the development of new biological data analysis tools in which the effort of the researcher is also used to build more sensible semantic models of organisms for the rest of the community. Building a model can be a time consuming and tedious task and may require domain experts, but in our opinion the effort is worth it, as it allows for the better annotation of data and therefore for the better interpretation of results. In addition, we believe that initiatives like the Open Biomedical Ontologies (OBO) [5,26] can benefit from Orymold and from applications like it since they bring the knowledge formalization discussion to the biological domain expert ground in an immediately useful way.

### Outlook

The next stages of development will include an open API to allow users to develop modules for deeper analysis of experimental data based on our application, and very probably to extend our annotation methods as more sources and web services become stabilized and available. It would be of great benefit to the community to implement a module to allow customized extraction of experimental MA data from standard databases such as GEO [27] and Array Express [28], which recently has released an alpha version of their web services API. Unfortunately, automatic retrieval of probe sequences from these databases is difficult, and in some cases, the expression value for a gene is calculated from a set of probe values. Uploading of this type of data goes against our preference to conserve the link with the original discrete data points measured in the experiment, as the choice of probes may influence the outcome of an experiment, and probes may even be wrongly or ambiguously assigned. In Orymold, the umbilical cord between what is measured (a probe for a well defined sequence) and the final data is never severed.

Presently, the Orymold application is restricted to performing simple queries, taking spatial and temporal localization of expression as the main parameters. It is our intention to develop modules to allow for more statistically sound analysis and data mining techniques. Although here we focussed on mRNA data, a module for protein data including IHC or proteomic data could be readily included, using BLAST to map epitopes, peptides, or proteins on the backend database in the same manner that probes were mapped for gene expression. Data from metabolomics experiments would also be interesting to include, yet systematic studies linking gene function with changes in metabolites are not yet available.

Finally, we would like also to extend the application to allow users to export their semantic models in a standard format, such as OBO and the Web Ontology Language (OWL) [29], so they contribute to the construction of standard ontologies.

## Conclusion

Orymold is a flexible and customizable software application for the integrative exploration of heterogeneous experimental data sources in a biologically meaningful context. Its architecture allows both individual and collaborative initiatives or research groups to rapidly and easily integrate experimental data in their own semantic model describing an organism, or part of an organism. An atlas of pictures coherent with the ontology can be included to improve the description of terms and to show experimental results. Once experimental data is integrated, it is straightforward to perform semantic queries and point out relevant trends on gene expression data based on both spatial and temporal expression patterns.

## Availability and requirements

• **Project name: **Orymold

• **Project home page: **

• **Operating system(s): **Platform independent

• **Programming language: **Java

• **Other requirements: **latest JRE, MySQL 5.0 or higher

• **License: **Proprietary license. Free for non-commercial purposes.

• **Any restrictions to use by non-academics: **licence needed.

## Abbreviations

API: (Application Programming Interface); FACS: (Fluorescent Activated Cell Sorting); GUI: (Graphical User Interface); IHC: (Immunohistochemistry); ISH: (*In Situ *Hybridization); JDBC: (Java Database Connectivity); JPEG: (Joint Photographic Experts Group); LAN: (Local Area Network); LCM: (Laser Capture Micro-dissection); MA: (Microarray); OBO: (Open Biomedical Ontologies); OWL: (Web Ontology Language); UML: (Unified Modelling Language); RDBMS: (Relational Database Management System); RMI: (Remote Method Invocation).

## Authors' contributions

JM designed and implemented the databases and client application and wrote the manuscript. AE supervised the design and implementation of both databases and the client application and helped writing the manuscript. JEA participated in the design and implementation of the client prototype. RA collected and processed the pictures of the atlas. JS tested the software and entered single probe-based data. TM conceived and directed the project and revised the manuscript. All authors read and approved the final manuscript.

## Supplementary Material

Additional file 1 **Diagram with alternative Orymold configurations**. This file contains two diagrams showing alternative client-server configurations for the Orymold system. Click here for file

Additional file 2 **Example of a semantic query result**. This file contains an example result of a semantic query performed using Orymold. Click here for file
