# Supplementary material for: Orymold: ontology based gene expression data integration and analysis tool applied to rice
Source: BMC Bioinformatics. 2009 May 23;10:158. doi: 10.1186/1471-2105-10-158 (PMC2696451; doi:10.1186/1471-2105-10-158)
Supplement: Additional file 1 — Diagram with alternative Orymold configurations. This file contains two diagrams showing alternative client-server configurations for the Orymold system. [file 1471-2105-10-158-S1.doc]

**Supplementary Figures. Alternative configurations of the system.**

**
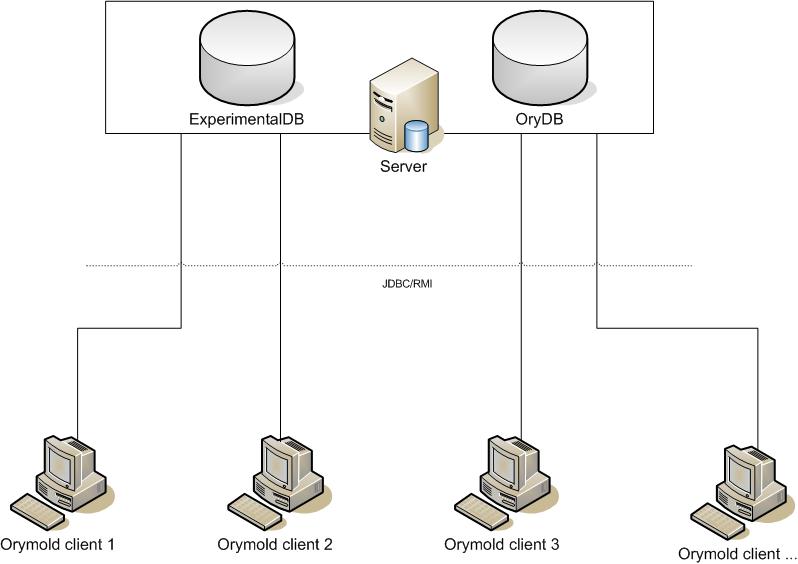
**

Configuration 1


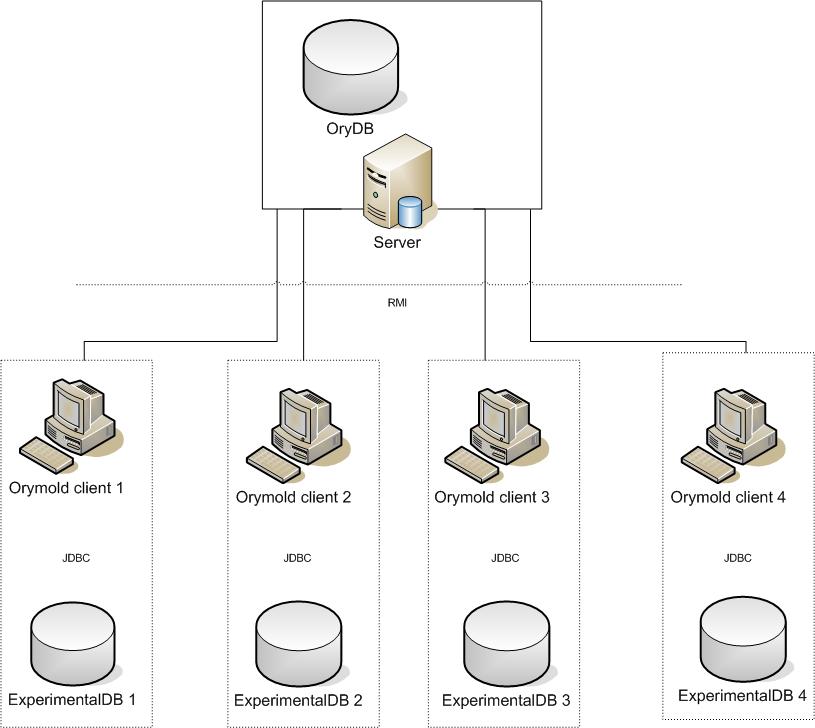


Configuration 2
