# Supplementary material for: Orymold: ontology based gene expression data integration and analysis tool applied to rice
Source: BMC Bioinformatics. 2009 May 23;10:158. doi: 10.1186/1471-2105-10-158 (PMC2696451; doi:10.1186/1471-2105-10-158)
Supplement: Additional file 2 — Example of a semantic query result. This file contains an example result of a semantic query performed using Orymold. [file 1471-2105-10-158-S2.pdf]

Supplementary File

Example of semantic queries use

A fast and simple method of using the tool is to try and find particular gene expression patterns according to the ontology. For instance, isolating genes expressed in a specific tissue or cell. This can be achieved using the basic query tools provided by Orymold. With them you can check the expression of a tissue or cell type against another tissue or cell type using the MINUS operation. MINUS operation retains all expressed genes of a region not expressed in the compared region. Doing this iteratively against all other tissues, organs and cell types (or a subset of them) allows building a refined list of interesting specific genes. We performed that process using bulliform cells and isolated a list of 72 tissue specific expressed genes. Most of them haven't been properly annotated yet and possess an 'hypothetical', 'expressed' or 'putative' label expressing the uncertainty of their function, which by itself suggests further research given their specificity. Among them, the one showing highest expression is labeled as “12010.m06222 auxin response factor 16, putative, expressed”. By consulting Orymold several other transcripts labeled as auxin response factors 16 are found to be expressed widely in the plant, but the selected transcript '12010.m06222' from the locus 'LOC_Os10g33940', is specifically expressed in the bulliform cells of the adult leaf's mid vein region, and its expression level is higher than that of any of the other putative auxin response factor 16 transcript in any other tested tissue. 12010.m06222 is homologuous to a gene which has recently been described in *A. thaliana* as a root cap formation and gravitropism related factor (Guilfoyle and Hagen 2007). In rice, several homologues, generated through gene duplication, have been identified (Wang et al. 2007), but their function remains to be assessed. The identification of 12010.m06222 points at a role of auxin in bulliform cells. Indeed, Fujino et al. recently described a rice mutant, *narrow leaf 7*, with specific alterations in bulliform cells associated with a reduction in IAA content. Thus Orymold rapidly pointed to 12010.m06222 as an interesting candidate gene for further analysis, which could be done through to the creation of knock out mutants for the gene to verify its function.

More complex searches can be performed with respect to combined patterns of expression among genes or plant regions using the advanced queries menu, which allows filtering of gene expression using thresholds and levels. Using advanced tools will lead to similar shortened lists, consequently leading to new research suggestion by iteratively constructing queries, checked through experimental data evidences. Recently, ArrayExpress launched their ATLAS Beta web based application (http://www.ebi.ac.uk/microarray-as/atlas/), which is intended to perform similar kind of queries on arrays. Nevertheless Orymold offers superior flexibility with respect to search parameter setting and the contextual support of a morphological atlas and ontology.

Example references

Guilfoyle T.J. and Hagen G., Auxin response factors, *Curr. Opin. Plant Biol.* **10** (2007), pp. 453–460.

Wang D., Pei K., Fu Y., Sun Z,., Li S., Liu H., Tang K., Han B. and Tao Y., Genome-wide analysis of the *auxin response factor* (ARF) gene family in rice (*Oryza sativa*), *Gene* **394** (2007), pp. 13–24.

Fujino K., Matsuda Y., Ozawa K., Nishimura T., Koshiba T., Fraaije MW, Sekiguchi H. NARROW LEAF 7 controls leaf shape mediated by auxin in rice. *Mol Genet Genomics*. 2008 May;279(5):499-507
